# Arsenic trioxide induces oxidative stress, DNA damage, and mitochondrial pathway of apoptosis in human leukemia (HL-60) cells

**DOI:** 10.1186/1756-9966-33-42

**Published:** 2014-05-16

**Authors:** Sanjay Kumar, Clement G Yedjou, Paul B Tchounwou

**Affiliations:** 1 Cellomics and Toxicogenomics Research Laboratory, NIH/NIMHD-RCMI Center for Environmental Health, College of Science, Engineering and Technology, Jackson State University, 1400 Lunch Street, Box18750, Jackson, Mississippi MS39217, USA

**Keywords:** Arsenic trioxide, Oxidative stress, DNA damage, Apoptosis, HL-60 cells

## Abstract

**Background:**

Acute promyelocytic leukemia (APL) is a subtype of acute myeloid leukemia (AML), which accounts for approximately 10% of all acute myloid leukemia cases. It is a blood cancer that is formed by chromosomal mutation. Each year in the United States, APL affects about 1,500 patients of all age groups and causes approximately 1.2% of cancer deaths. Arsenic trioxide (ATO) has been used successfully for treatment of APL patients, and both induction and consolidated therapy have resulted in complete remission. Recently published studies from our laboratory have demonstrated that ATO pharmacology as an anti-leukemic drug is associated with cytotoxic and genotoxic effects in leukemia cells.

**Methods:**

In the present study, we further investigated the detailed molecular mechanism of ATO-mediated intrinsic pathway of apoptosis; using HL-60 cells as a test model. Oxidative stress was assessed by spectrophotometric measurements of MDA and GSH levels while genotoxicity was determined by single cell gel electrophoresis (Comet assay). Apoptosis pathway was analyzed by Western blot analysis of Bax, Bcl2 and caspase 3 expression, as well as immunocytochemistry and confocal imaging of Bax and Cyt c translocation and mitochondrial membrane potential depolarization.

**Results:**

ATO significantly (*p* < 0.05) induces oxidative stress, DNA damage, and caspase 3 activityin HL-60 cells in a dose-dependent manner. It also activated the intrinsic pathway of apoptosis by significantly modulating (*p* < 0.05) the expression and translocation of apoptotic molecules and decreasing the mitochondrial membrane potential in leukemia cells.

**Conclusion:**

Taken together, our research demonstrated that ATO induces mitochondrial pathway of apoptosis in HL-60 cells. This apoptotic signaling is modulated via oxidative stress, DNA damage, and change in mitochondrial membrane potential, translocation and upregulation of apoptotic proteins leading programmed cell death.

## Background

Acute promyelocytic leukemia (APL) is a subtype of acute myeloid leukemia (AML), which causes approximately 1.2% of cancer deaths in USA [1,2]. APL is a blood cancer that affects all age groups of people and strikes about 1,500 patients in the United States each year [3]. Initially, APL was treated with conventional chemotherapy method by using cytarabine and daunorubicin to achieve complete remissions (CRs) in approximately 70% of patients having 5-year disease-free survival of 35–45% [4,5]. All trans retinoic acid (ATRA) has brought revolutionary change for APL patients treatment. Combination of ATRA plus an anthracycline, with or without cytarabine achieved remission rates of nearly 90% for APL patients [1]. Although many therapeutic advances such as combined chemotherapy and hematopoietic stem cell transplantation have been made to improve the survival rate of APL patients, a higher proportion of patients relapse and hence do not undergo complete remission. Also, because of the growing evidence of resistance to ATRA treatment of APL patients [6], the U.S. Food and Drug Administration (FDA) approved arsenic trioxide (ATO) for APL patient treatment in September 2000 on the basis of several human clinical trials showing very promising results [7].

ATO is a drug of choice for the treatment of both relapsed and refractory APL patients. It is used alone or combination with all trans retinoic acid (ATRA) to achieve complete remission and maximum survival rate [8,9]. Existing evidence has shown that APL patients treated with ATO achieved complete remission with high survival rate without ATRA combination [10]. In a Phase II clinical trial study, it was reported that these APL patients treated with ATO alone observed a high rate of 5-years disease free survival (DFS) and an overall survival (OS) [11]. Few reports have suggested that ATO inhibits proliferation of human myeloma cells by cell cycle arrest [12] and induces apoptosis in HL-60 cells by phosphotidylserine externalization as well as DNA laddering [3].

ATO is a clastogenic/genotoxic compound. It has been shown to induce DNA damage/mutation in cultured mouse lymphoma cells [13] and bone marrow cells of Sprague–Dawley rats [14]. It induces DNA damage through double strands break in human colon cancer cells [15] and also DNA fragmentation as well as p53 activation in gastric cancer cells [16]. ATO also modulates stress gene (p53) expression in human liver carcinoma cells (HepG2) [17].

Although the detailed molecular mechanisms of the anti-cancer potency of ATO are not well understood, it has been shown to induce oxidative stress in hepatocellular carcinoma cells [18] and apoptosis in leukemia as well myeloma cells [19,20]. It has also been reported to induce apoptosis in cancer cells through cell cycle arrest [21] and modulation of apoptotic genes expression in NB4 cells [22]. ATO has also been shown to induce mitotic arrest and apoptosis in NB4 cells by changing mitochondrial membrane potential [23]. However, the detailed molecular mechanisms of ATO-induced oxidative stress, genotoxicity, and intrinsic pathway of apoptosis in HL-60 cells are not well elucidated. Therefore, in the present study, we investigated ATO–induced oxidative and genotoxic stress and its resulting impact on specific biomarkers of the mitochondrial pathway of apoptosis inhuman leukemia (HL-60) cells. HL-60 cell line has been derived from peripheral blood leukocytes of a patient with acute promyelocytic leukemia [24].

## Methods

### Cell line and culture

The APL cell line used in this study was HL-60. The Cells were purchased from the American Type Culture Collection (Manassas, VA), and maintained at 37°C in an atmosphere of 5% CO2 and 95% air according to standard procedures. HL-60 cells were cultured in Iscove’s Modified Dulbecco’s Medium (IMDM) containing 10% fetal bovine Serum (FBS) and 1% penicillin-streptomycin solution with cell density, 2×10^5^ viable cells/ml. 5×10^7^ cells were seeded for each dose of arsenic trioxide and incubated 24 hour at 37°C inside C0_2_ incubator.

### Chemicals and reagents

ATO was purchased from Fischer Scientific (Pittsburgh, PA). Mitochondrial isolation kit, Caspase assay kit, protease inhibitor and Glutathione assay kit were obtained from Sigma-Aldrich (St. Louis, MO). Anti-Cytochrome C, anti-Bax and anti-Bcl2 were purchased by Cell Signaling Technology (Danvers, MA). Lipid peroxidation kit and caspase 3 kit were obtained from Abcam (Cambridge, MA). Mitotracker red, Hoechst 33342, Alexa fluor 568 and Alexa fluor 568 were purchased from Life Technologies (Grand Island/NY).

### Measurement of reduced GSH

Leukemia cells were grown in presence or absence of ATO and the GSH content inside the cytoplasm was measured following a previously published protocol [25].

### Lipid peroxidation assay

HL-60 cells were treated with or without ATO and lipid peroxidation was evaluated by measuring malondialdehyde (MDA) levels using the lipid peroxidation assay kit (Abcam) as previously described [25].

### Single cell gel electrophoresis (Comet) assay

HL-60 cells were cultured in presence or absence of ATO and DNA damage was analyzed by performing a very sensitive alkaline comet assay as previously described [26], with few modifications in our laboratory [27,28]. The whole process was carried out under yellow light in order to minimize UV light damage. Agarose was prepared through melting in a boiling water bath and allowing it to return to room temperature. The cells were mixed with the melted agarose in a 1:10 ratio. Approximately 75 μL of the mixture of agarose and cells were placed on comet slides, and the agarose was solidified at 4°C for 10 min. After 10 min, the slides were placed in a lysis solution at 4°C for 30 min to lyse the embedded cells in the agarose. The excess lysis solution was removed from the slides and placed in an alkaline solution to denature the DNA for 40 min at room temperature. Later, the slides were subjected to TBE (Tris borate EDTA buffer) electrophoresis for 10 min with 1 volt/cm current between the two electrodes. Then the slides were fixed with 70% ethanol for 5 min, followed by SYBR green staining. The stained slides were examined using an epifluorescent microscope (Olympus BX51 TRF, USA). The data were analyzed with DNA damage analysis software (Loats Associates Inc., USA). The control comet slides were prepared along with the test comet slides under yellow light

### Western blotting analysis

Western blot analysis was conducted to determine specific cellular responses targeting apoptosis-related proteins including Bax, cyt C and Bcl-2. HL-60 cells were treated with different doses of ATO for 24 hr at 37°C. After incubation, cells were washed twice with cold phosphate buffered saline (PBS) and lysed in RIPA buffer containing (1% Nonidet P-40, 0.5% sodium deoxycholate, 0.1% SDS, 100 μg/ml phenylmethylsulfonyl fluoride, 100 μg/ml aprotinin, 1 μg/ml leupeptin, and 1 mm sodium orthovanadate) on ice 20 min. It was centrifuged at 14000 rpm for 12 min and supernatant collected in fresh micro centrifuge tubes. The total protein of cells extracts contained in the supernatant was measured by the Bradford method at 595 nm using a microtiter plate reader [29]. An equal amount (40 μg) of protein from control or treated cells was loaded per lane on a 10% SDS-PAGE gel, transferred into nitrocellulose membrane and analyzed by Western blotting for each specific protein of interest using its specific antibody as described previously [30]. The band intensities were quantified using Image J (National Institutes of Health).

### Confocal microscopy for Bax and Cytochrome c translocation

HL-60 cells (1×10^6^ cells) were grown in presence or absence of ATO and further incubated with mitotracker Red CMXRos (250 nM) for 30 min in dark at 37°C to stain mitochondria. After staining, cells were washed twice with PBS and adhered on poly- L- lysine coated chambered slide. Cells were fixed by adding 3% paraformaldehyde solution and permeabilized with 0.2% Nonidet P-40 in PBS containing glycine (0.5%). Cells were blocked in PBS containing 3% BSA for 30 min, then incubated with cytochrome C antibody (1:100 dilution) at 4°C overnight. Cells were washed with PBS and incubated with Alex fluor 568 tagged secondary Ab (1:1000) for 1 h at 4°C in dark. The cells were then incubated with Hoechst 33342 with (1:1000) for 7 min in dark to stain the nucleus. The cover slips were then mounted on slides using 90% glycerol containing 0.025% PPD as antifade. The images were acquired using the confocal microscope (Olympus Company, Center valley, PA) at appropriate excitation (578 nm) and emission (603 nm) wavelengths.

### Caspase -3 activity assay

Caspase-3 activity was measured in cytosolic fraction of control and ATO-treated HL-60 cells, using commercially available kits and according to manufacturer protocol (Sigma, St. Louis, MO, USA). In brief, cytosolic fraction of cells from both control and ATO treated was prepared as described earlier [31]. Equal amount of cytosolic proteins were used for the assay of caspase 3 activity. Cytosolic protein (50 μg) was mixed in a microtiter plate with assay buffer and caspase specific substrates (Ac-DEVD-pNA for caspase-3). After 4–16 h incubation at 37°C, the absorbance of pNA released as a result of caspase-3 like activity was measured at 405 nm in a microtiter plate reader as described in technical bulletin. The absorbance of negative control (assay buffer substrate) was subtracted from specific values. Mean values of triplicate measurements were presented.

### Measurement of change in mitochondrial membrane potential (Δψm)

The integrity of the inner mitochondrial membrane may be measured by observing the potential gradient across this membrane. This can be achieved by measuring the uptake of the cationic carbocyanine dye, JC1 into the matrix. Mitochondria were isolated from control and ATO-treated HL-60 cells using mitochondria isolation kit (Sigma, St. Louis, MO, USA). Isolated mitochondria were incubated with 2 μl JC1 stain (from stock 1 mg/ml) and 950 μl JC1 assay buffer for 10 min in dark at 25°C. The fluorescence of each sample (total assay vol. 1 ml) was recorded using a Perkin Elmer LS50B spectrofluorometer (excitation 490 nm, slit, 5 nm; emission 590 nm, slit, 7.2 nm) [32].

### Immunocytochemistry

HL-60 cells (1×10^5^) were cultured in presence or absence of ATO and placed on poly-L-lysine coated slide. Cells were fixed by using 3% paraformaldehyde and permeablized with 0.2% NP-40 containing 0.5% glycine. After blocking with 4% BSA, fixed cells were incubated overnight with Ki-67 antibody (dilution, 1:100) (cat# 33–4711) from life technology company at 4°C. After incubation, cells were washed with PBS three times and tagged with secondary antibody (anti-mouse fluorescein) for one hour at room temperature followed by Hoechst 33342 (dilution, 1:2000) staining 7 min. Slides were washed with PBS and paste coverslip using prolong gold antifade reagent. After drying, slides were imaged by confocal microscopy (Olympus company, Center valley, PA).

### Statistical analysis

Experiments were performed in triplicates. Data were presented as means ± SDs. Where appropriate, one-way ANOVA or student paired *t*-test was performed using SAS Softwareavailable in the Biostatistics Core Laboratory at Jackson State University. p-values less than 0.05 were considered statistically significant.

## Results

### Arsenic trioxide induces oxidative stress in Hl-60 cells

**Figure 1:**
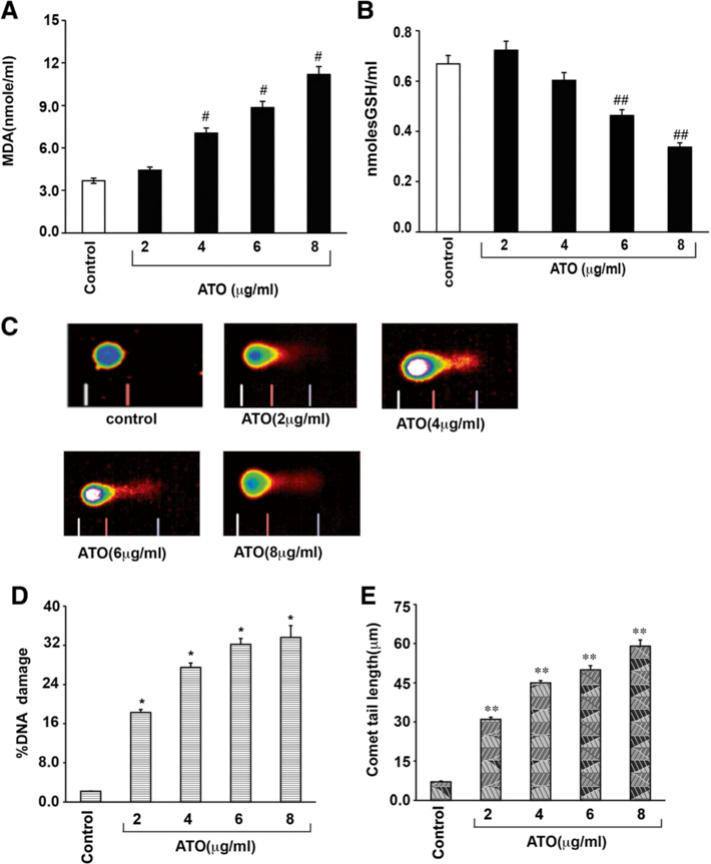
**Arsenic trioxide induces oxidative stress in HL-60 cells. (A)** HL-60 cells were incubated with 2, 4, 6 and 8 mg/ml of ATO for 24 hrs and the level of malondialdehyde(MDA) was measured by spectrophotometry at 532 nm. MDA was expressed in nmole/ml. Data represent the means of three independent experiments ± SDs (# P < 0.05). **(B)** Cells were treated with different doses of ATO for 24 hrs and reduced GSH level was measured by spectrophotometry at 412 nm. GSH was expressed in nmole GSH/ml. Data represent the means of three independent experiments ± SDs (##P < 0.05). **(C)** HL-60 cells were grown in absence or presence of different doses of ATO for 24 hrs and DNA damage was analyzed by alkaline Comet assay. **(D)** ATO – induced genotoxicity was expressed as percentage of DNA damage. Data represent the means of three independent experiments ± SDs (**P < 0.01). **(E)** ATO-induced comet tail length was measured in micrometer. Data represent the means of three independent experiments ± SDs (***P < 0.01).

In the present study we investigated three biomarkers of oxidative stress including lipid peroxidation as characterized by malondialdehyde (MDA) production, cellular GSH content, and DNA damage in HL-60 cells following treatment with different doses of ATO. Interestingly, ATO treatment significantly increased MDA level (Figure 1A) as well as percentages of DNA damage and Comet tail length (Figure 1C-E) in a dose- dependent manner. Contrary, a significant decrease in GSH content was observed at higher level of ATO exposure (Figure 1B).

### Arsenic trioxide modulates apoptotic proteins expression

**Figure 2:**
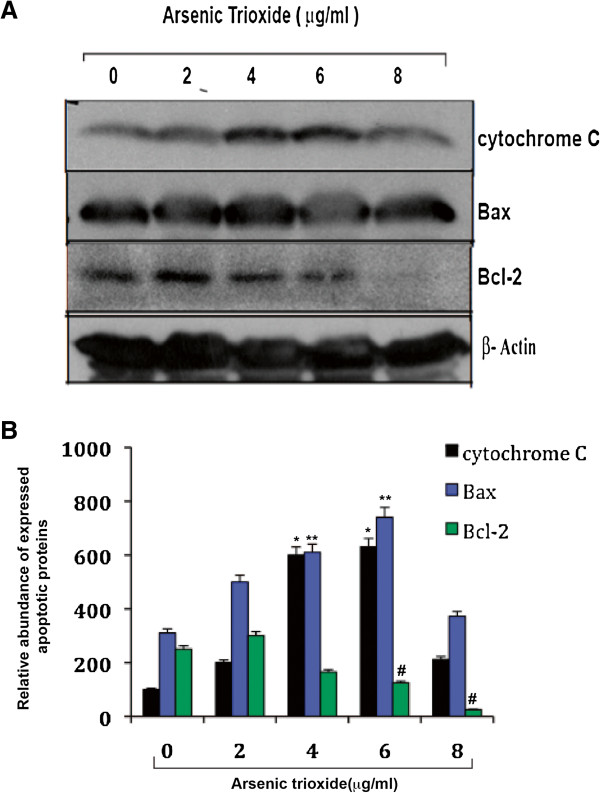
**Arsenic trioxide modulates apoptotic proteins expression. (A)** Western blots of intrinsic apoptotic pathway proteins in control and ATO-treated HL-60 cells. ATO exposure significantly increased the expression levels of Bax, cytochrome C, and decreased the expression level of Bcl-2 in a dose- dependent manner. **(B)** Densitometric analysis of ATO –induced apoptotic proteins expression in HL-60 cells. Data represent the means of three independent experiments ± SDs (*p < 0.01; **p < 0.05 and #*p* < 0.01).

ATO-induced oxidative stress in HL-60 cells also caused an increase in the expression level of pro-apoptotic proteins (Bax and cytochrome C) and reduced the expression level of anti-apoptotic protein (Bcl-2), in a dose-dependent manner (Figure 2A). Densitometric analysis has shown that ATO-induced apoptotic proteins, cytchrome C and Bax expression significantly (*p* < 0.05) increased at 4 and 6 μg/ml ATO treated HL-60 cells lysate (2B). Whereas, anti-apoptotic protein, Bcl-2 expression was significantly down regulated at 6 and 8 μg/ml ATO treatment cells lysate (2B).

### Arsenic trioxide changes inner mitochondrial membrane potential

**Figure 3:**
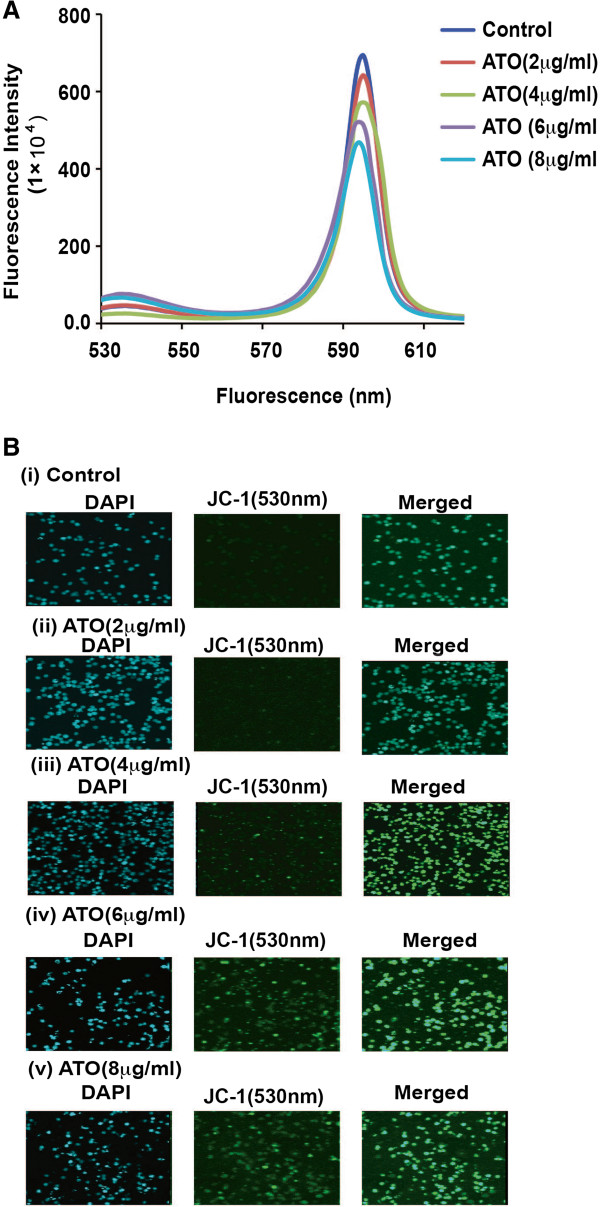
**ATO changes mitochondrial membrane potential (Δψm). (A)** ATO treatment was changed the mitochondrial membrane potential in a dose- dependent manner. [**(B)(i-v)**] There are three subsets of each treatment-DAPI (blue), JC-1 monomer (excitation 530 nm, green) and merged (blue/green). ATO treatment dose–dependently changed mitochondrial membrane potential and opened transition pores. It helped to release J-aggregate and continuously increased JC-1 monomer (green color) in a dose dependent manner in HL-60 cells.

Due to imbalance ratio of Bax and Bcl-2 protein expression, ATO treatment lead to change in inner mitochondrial membrane potential and opening of transition pores. We have measured this change in mitochondrial membrane potential after treatment of cells with different doses of ATO and by labeling with very sensitive cationic carbocynine dye, JC-1. In control sample, healthy mitochondria showed high mitochondrial membrane potential (ψm) with intact membrane and accumulated in their matrix more JC-1 to form J- aggregates, showing intense fluorescence at 590 nm. Whereas in ATO treated cells, mitochondria showed lower ψm and less accumulation of JC-1 in their matrix leading to less formation of J-aggregates, and weak fluorescence at 590 nm (Figure 3A). We have also done confocal microscopy imaging of control and ATO-treated cells followed by staining with JC-1 and DAPI. JC-1 monomer (530 nm) expression was activated by ATO treatment in a dose-dependent manner [Figure 3B (i-v)].

### Arsenic trioxide stimulates translocation of Bax and Cytochrome C

**Figure 4:**
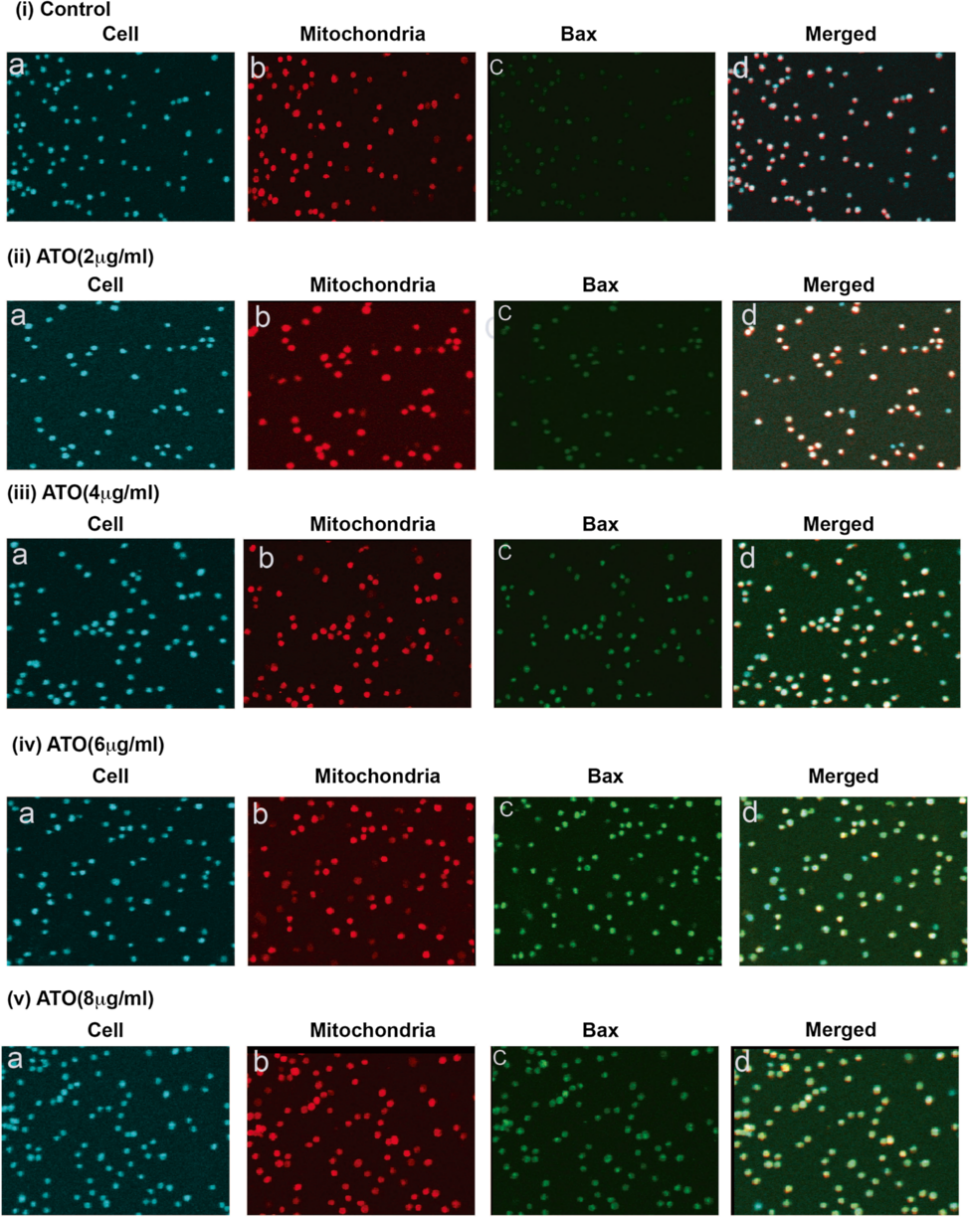
**(i-v) Arsenic trioxide stimulates translocation of Bax protein.** Each image set contains four subsets, a - cells stained with DAPI (blue); b – mitochondria stained with mitotracker red CMXRos (red, 250 nM); c – Bax protein tagged with fluorescent secondary antibody (green); and d – merged image of all previous three (a, b and c). Both immunocytochemistry and confocal imaging show translocation of pro-apoptotic protein, Bax from cytosol to mitochondria in a dose – dependent manner.

**Figure 5:**
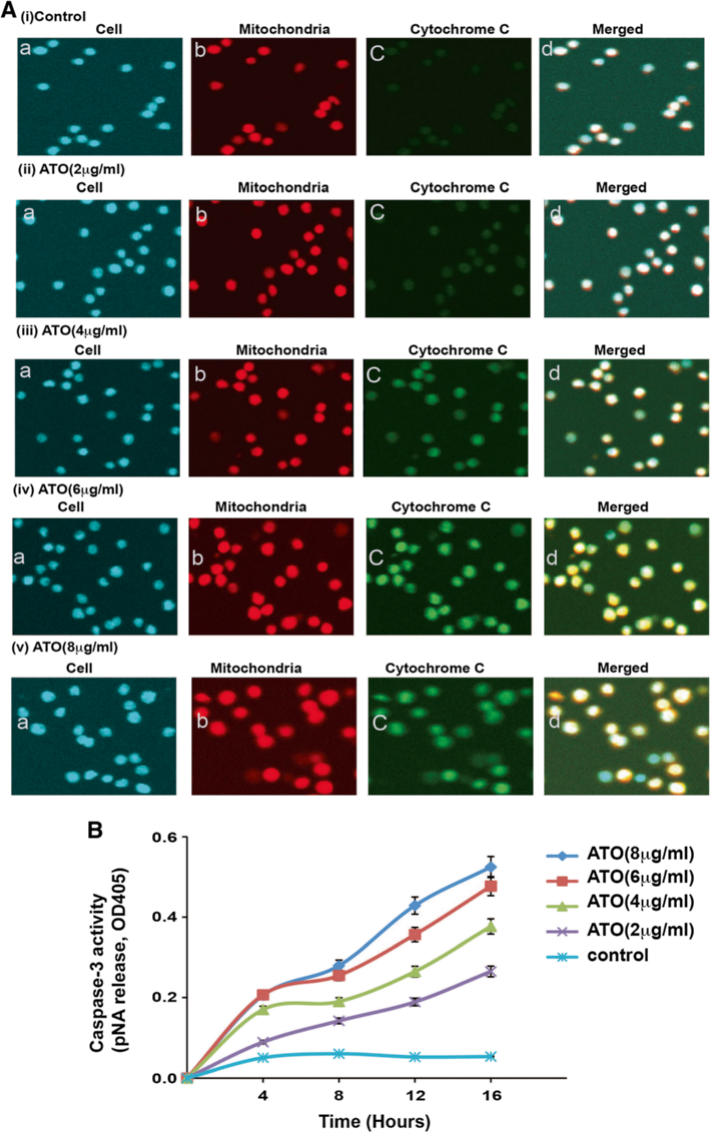
**Arsenic trioxide induces release of cytochrome C protein from mitochondria and activation of caspase 3. [(A) (i-v)]** Each set contains four subsets, a – cells stained with DAPI (blue); b- mitochondria stained with mitotracker red CMXRos (red, 250nM); c –cytochrome C protein tagged with fluorescent secondary antibody (green); and d – merged image of all previous three (a, b and c). Here, it is shown that cytochrome C was released from mitochondria in a dose- dependent manner. **(B)** Data also show a dose-dependent enhancement of caspase 3 activity with the ATO treatment of HL-60 cells.

Previous research has reported that oxidative stress activates translocation of pro-apoptotic proteins from cytosol to mitochondria and release of cytochrome C from mitochondria to cytoplasm inside cell [33]. We have checked ATO-induced translocation of pro-apoptotic protein, Bax from cytosol to mitochondria and cytochrome C from mitochondria to cytosol by labeling cells with Hoechst staining, mitochondria with mitotracker red and Bax as well as cytochrome C protein with green fluorescent antibody. Our results show that the amount of translocated Bax inside mitochondria [Figure 4 (i-v)] and cytochrome C protein in cytosol of ATO treated HL-60 cells increased in a dose-dependent manner [Figure 5A (i-v)]. We used green fluorescent tag anti-Bax and anti-cytochrome C antibody to recognize translocation of Bax and cytochrome C by immunocytochemistry and confocal imaging of cells.

### Arsenic trioxide stimulates Caspase-3 activity

Inside the cytosol, cytochrome C stimulates a series of apoptotic signaling molecules along with variety of caspases (like caspase 9) and finally caspase3 which is main executioner of mitochondrial pathway of apoptosis [34]. We have investigated the caspase 3 activity in HL-60 cells following treatment with different doses of ATO. Interestingly, ATO upregulatedcaspase 3 activity in a dose-dependent manner (Figure 5B).

## Discussion

Previous studies have reported that ATO diffuses through cell membrane into the cytoplasm and produces cytotoxic effect by generating reactive oxygen species. It has also been reported that ATO causes oxidative stress and cell death in a variety of cells including acute promyelocyte leukemia (APL), acute myeloid leukemia and chronic myeloid leukemia as well as solid tumor cells *in vitro*[35], but leukemia cells appear to be more susceptible and clinical important than others [36]. Earlier studies have also pointed out that lower doses of ATO induce cell proliferation, while higher doses inhibit growth in NB4 as well as lymphoid malignant cells [21,37]. ATO has also been found to inhibit DNA synthesis in human colon cancer cells [15] and proliferation in myeloma cell lines dose –dependent manner [12]. Recently, several groups have provided evidence that ATO induces cell cycle arrest and apoptosis in a variety of leukemia as well as myeloma cells [12,38]. But the detailed mechanisms of toxicity to HL-60 cells mostly remain unknown. Here, we have elucidated the molecular mechanisms ATO-induced oxidative stress and intrinsic pathway of apoptosis in HL-60 cells. Our findings indicate that ATO causes oxidative stress through generation of ROS, increase in lipid peroxidation, induction of DNA damage and reduction of GSH level in HL-60 cells (Figure 1A-E).

Accumulating data have suggested that ATO - induced apoptosis is associated with down-regulation of Bcl-2 protein in NB4 cells [22] and activation of Bax protein expression as well as reduction of mitochondrial membrane potential in lymphoma B-cells [39]. Our data presented here reveal that ATO activated Bax and cytochrome C expression and down-regulated Bcl-2 protein expression in HL-60 cells in a dose-dependent manner (Figure 2A & B). ATO-induced oxidative stress and alteration of Bax and Bcl-2 proteins expression lead to change in mitochondrial membrane potential of HL-60 cells. In ATO-treated cells, we found that a significant decrease in mitochondrial membrane potential and increase in JC-1-monomer (green color) in a dose-dependent manner (Figure 3A-B). It has also been reported from other studies that oxidative stress stimulates translocation of Bax from cytosol to mitochondria and release of cytochrome C inside cytoplasm during liver apoptosis [33]. Other research groups have reported that ATO-induced apoptosis is associated with Bax translocation in cervical cancer cells [40], and release of cytochrome C from mitochondria in lymphoma B-cells [39]. Our results support these findings showing that ATO induces translocation ofBax and cytochrome in HL-60 cells a dose-dependent manner [Figure 4 (i-v) and 5A (i-v)]. Inside the cytosol, cytochrome C seems to activate different signaling molecules along with a variety of caspases and finally caspase 3 in the intrinsic pathway of apoptosis.

**Figure 6:**
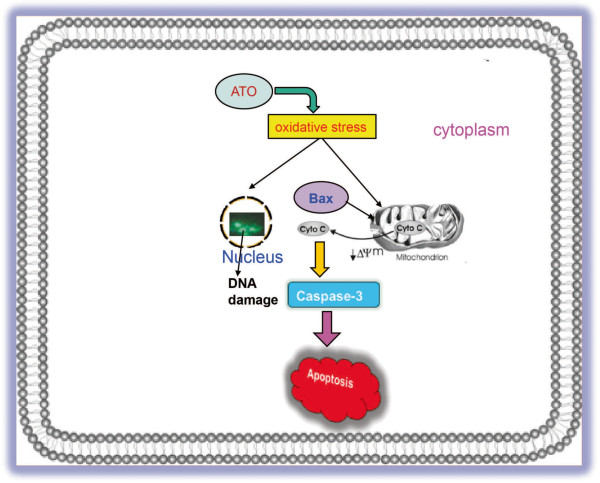
**ATO-induced intrinsic pathway of apoptosis in HL-60 cells.** ATO induces oxidative stress in APL cells through lipid peroxidation, GSH content changed and DNA damage. It changes mitochondrial membrane potential and modulates expression and translocation of apoptotic proteins, which lead to caspase3 activity and apoptosis in HL-60 cells.

Other studies have demonstrated the role of caspase 3 in chemical-induced apoptosis. Cellfood™ induces apoptosis in leukemia cell lines (U937, Jurkat) through caspase-3 activation and DNA fragmentation [41]. Cinnamic acid also causes apoptosis in melanoma cells (HT-144) by caspase-3 activation and DNA damage [42]. Baicalin induces intrinsic pathway of apoptosis in lymphoma cells via DNA fragmentation, modulation of apoptotic and caspase-3 proteins expression [43]. Interestingly, we found that ATO treatment increased caspase 3-activity in a dose-dependent manner (Figure 4B). ATO as a genotoxic compound induces clastogenic effect in HL-60 cells through oxidative DNA damage and oxidative stress in a dose dependent manner. ATO has been reported to inhibit unscheduled DNA synthesis in V79 Chinese hamster cells by excision of pyrimidine dimmers [44]. Erlotinib, an inhibitor of EGFR enhances ATO mediated DNA double –strand break/damage by preventing EGFR –mediated DNA double-strand break repair human A549 lung cancer cells [45]. ATO – induced oxidative stress produces epigenetic effect through specific DNA base modification on exposure of mammalian cells and production of 8-hydroxy-2'-deoxyguanosine (8-OHdG) [46]. It is shown to increase oxidative DNA damage product, 8-OHdG in acute promyelocytic leukemia patients during arsenic therapy [47]. ATO causes apoptosis in multiple myeloma cells by disruption of mitochondrial membrane potential and caspase-3 activity [48]. It also induces apoptosis in lymphoid neoplasms through cell cycle arrest [21,49], as well as in plasma cells from myeloma patients [50]. ATO induces apoptosis in NB4 cells through down-regulation of Bcl-2 expression and modulation of PML-RARα/PML proteins [22]. Similar to Domoic acid and Okadaic acid (natural toxicants) [51], ATO bears both genotoxic and epigenetic properties. Taken together, we have demonstrated from our research that ATO induces mitochondrial pathway of apoptosis through oxidative stress; modulating expression and translocation of apoptotic proteins, and changing inner mitochondrial membrane potential and caspase 3 activity in HL-60 cells (Figure 6).

## Conclusions

It can be concluded from the present *in vitro* study that arsenic trioxide induces mitochondrial pathway of apoptosis in HL-60 cells. Although the exact anti-leukemic molecular mechanism of ATO is not well understood, we have investigated in present study its detailed mechanism of oxidative stress-induced intrinsic pathway of apoptosis by modulation of expression and translocation of apoptotic proteins, changing mitochondrial membrane potential and activation of caspase 3 activity in HL-60 cells. By elucidating the anti-leukemic mechanisms of action of ATO in HL-60 cells, we are able to provide new insights into the molecular targets, and a rational basis for drug designing for a more prominent APL chemotherapy in the future.

## Abbreviations

ANOVA: One way analysis of variance; APL: Acute promyelocytic leukemia; ATO: Arsenictrioxide; ATRA: All trans retinoic acid; DMSO: Dimethylsulfoxide; DNA: Deoxyribonucleic acid; FACS: Fluorescenceactivated cell sorting system; MDA: Malondialdehyde; MTT: 3-(4,5-dimethyl-2-thiazolyl)-2,5-diphenyl-2tetrazoliumbromide; PBS: Phosphate buffer saline; ROS: Reactive oxygen species.

## Competing interests

The authors declare that they have no competing interests.

## Authors’ contributions

SK and PBT conceived, designed and implemented the study, and drafted the manuscript.CGY participated in the implementation of research activities. All authors read and approved the final draft of the manuscript.
